# A new class of Poisson Ridge-type estimator

**DOI:** 10.1038/s41598-023-32119-0

**Published:** 2023-03-27

**Authors:** Esra Ertan, Kadri Ulaş Akay

**Affiliations:** Department of Mathematics, Science Faculty, University of Istanbul, Vezneciler, Beyazit, 34134 Istanbul, Turkey

**Keywords:** Statistics, Mathematics and computing

## Abstract

The Poisson Regression Model (PRM) is one of the benchmark models when analyzing the count data. The Maximum Likelihood Estimator (MLE) is used to estimate the model parameters in PRMs. However, the MLE may suffer from various drawbacks that arise due to the existence of multicollinearity problems. Many estimators have been proposed as alternatives to each other to alleviate the multicollinearity problem in PRM, such as Poisson Ridge Estimator (PRE), Poisson Liu Estimator (PLE), Poisson Liu-type Estimator (PLTE), and Improvement Liu-Type Estimator (ILTE). In this study, we define a new general class of estimators which is based on the PRE as an alternative to other existing biased estimators in the PRMs. The superiority of the proposed biased estimator over the other existing biased estimators is given under the asymptotic matrix mean square error sense. Furthermore, two separate Monte Carlo simulation studies are implemented to compare the performances of the proposed biased estimators. Finally, the performances of all considered biased estimators are shown in real data.

## Introduction

The Poisson Regression Model (PRM) is one of the benchmark models for count data in much the same way as the normal linear regression model is the benchmark for continuous data^1^. In the PRM, $y_{i}$ is the response variable and follows a Poisson distribution with mean $\mu_{i}$, then the probability function is defined as

1 $$f\left( {y_{i} } \right) = \frac{{e^{{ - \mu_{i} }} \mu_{i}^{{y_{i} }} }}{{y_{i} !}}, \quad i = 1,2, \ldots ,n, y_{i} = 0,1,2, \ldots$$

where $\mu_{i}$ is expressed by using canonical log link function and a linear combination of explanatory variables as follows $\mu_{i} = \exp \left( {x^{\prime}_{i} \beta } \right)$ where $x^{\prime}_{i}$ is the *i*th row of *X*, which is an $n \times \left( {p + 1} \right)$ data matrix with *p* explanatory variables and $\beta$ is a $\left( {p + 1} \right) \times 1$ vector of coefficients.

The Maximum Likelihood method is the well-known estimation technique to estimate the model parameters in PRMs^2^. The log-likelihood function for PRM is given as follows

2 $$l(\beta ) = \sum\limits_{i = 1}^{n} {y_{i} x^{\prime}_{i} \beta - \exp \left( {x^{\prime}_{i} \beta } \right) - \log \left( {y_{i} !} \right).}$$

The Maximum Likelihood Estimator (MLE) of $\beta$ is obtained by maximizing the log-likelihood function, so the following equations are obtained as

3 $$S(\beta ) = \frac{\partial l(\beta ;y)}{{\partial \beta }} = \sum\limits_{i = 1}^{n} {\left[ {y_{i} - \exp \left( {x^{\prime}_{i} \beta } \right)} \right]} x_{i} = 0.$$

Since Eq. (3) is nonlinear function of parameter $\beta$, the solution of $S\left( \beta \right)$ is obtained using the following iteratively reweighted least squares (IRLS) algorithm

4 $$\hat{\beta }_{MLE} = \left( {X^{\prime}\hat{W}X} \right)^{ - 1} X^{\prime}\hat{W}Z,$$

where Z is an *n*-dimensional vector with the *i*th element $z_{i} = \log \left( {\hat{\mu }_{i} } \right) + \frac{{y_{i} - \hat{\mu }_{i} }}{{\hat{\mu }_{i} }}$ and $\hat{W} = {\text{diag}} \left[ {\hat{\mu }_{i} } \right]$^3^. The iteration ends when the difference between the old and updated values is less than a given small value, which is usually $10^{ - 8}$^4^. The asymptotic variance–covariance matrix of $\hat{\beta }_{MLE}$ is $cov\left( {\hat{\beta }{}_{MLE}} \right) = \left( {X^{\prime}\hat{W}X} \right)^{ - 1} .$

Besides being a widely used estimator of MLE, one of its major disadvantages is that parameter estimates become unstable in the case of multicollinearity^5–13^. The multicollinearity problem, which occurs because of the approximately linear relationship between the explanatory variables, affects the estimates of model parameters in the PRMs as well as in the linear regression models. One effect of the multicollinearity between explanatory variables is that the variance of the MLE becomes so large that the estimates of the model parameters become unstable^14–20^.

In order to reduce the undesirable effects of multicollinearity, the biased estimators that are alternative to the MLE are generalized in a manner similar to that introduced in the linear regression model. For example, Månsson and Shukur^18^ proposed the Poisson Ridge Estimator (PRE) as follows:

5 $$\hat{\beta }_{PRE} = \left( {X^{\prime}\hat{W}X + kI} \right)^{ - 1} X^{\prime}\hat{W}X\hat{\beta }_{MLE} {, }\quad k > 0,$$

where $k$ is a biasing parameter. The PRE is the generalization of the Ridge estimator introduced by Hoerl and Kennard^21^ for the linear regression model.

Månsson et al.^19^, Amin et al.^22^ and Qasim et al.^23^ defined the Poisson Liu Estimator (PLE) as follows:

6 $$\hat{\beta }_{PLE} = \left( {X^{\prime}\hat{W}X + I} \right)^{ - 1} \left( {X^{\prime}\hat{W}X + dI} \right)\hat{\beta }_{MLE} ,$$

where $0 < d < 1$ is a biasing parameter. The PLE is the generalization of the Liu estimator introduced by Liu^24^ for the linear regression model.

In recent years, the estimators with two biasing parameters have been proposed as an alternative to PRE and PLE. The purpose of estimators with two biasing parameters obtained by combining several estimators is to obtain more suitable estimators for parameter estimates. In this context, Algamal^25^ defined the Poisson Liu-type estimator (PLTE) for the PRMs as follows:

7 $$\hat{\beta }_{PLTE} = \left( {X^{\prime}\hat{W}X + kI} \right)^{ - 1} \left( {X^{\prime}\hat{W}X - dI} \right)\hat{\beta }_{MLE} ,$$

where $k{ > 0}$ and $d \in R$ are the biasing parameters. The PLTE is a generalization of the Liu-type estimator, which is firstly introduced by Liu^26^. The PLTE is based on the biasing parameters $k$ and $d$.

Moreover, Asar and Genç^15^ and Çetinkaya and Kaçıranlar^16^ proposed another biased estimator with two biasing parameters, defined by Özkale and Kaçıranlar^27^ for the linear regression models. The Poisson two-parameter Estimator (PTPE) is defined as:

8 $$\hat{\beta }_{PTPE} = \left( {X^{\prime}\hat{W}X + kI} \right)^{ - 1} \left( {X^{\prime}\hat{W}X + kdI} \right)\hat{\beta }_{MLE} ,$$

where $k{ > 0}$ and $0{ < }d{ < 1}$ are the biasing parameters.

As an alternative to the estimators introduced so far, Akay and Ertan^5^ proposed a general Improved Liu-type Estimator (ILTE) which includes MLE, PRE, PLE, PLTE and PTPE as special cases as follows:

9 $$\hat{\beta }_{ILTE} = \left( {X^{\prime}\hat{W}X + kI} \right)^{ - 1} \left( {X^{\prime}\hat{W}X + f\left( k \right)I} \right)\hat{\beta }{\kern 1pt}^{*} , k > 0,$$

where $\hat{\beta }^{*}$ is any estimator of $\beta$ and $f\left( k \right)$ is a continuous function of the biasing parameter *k*. The estimator given in (9) is a generalization of the Liu-type estimator proposed by Kurnaz and Akay^28^ for linear regression models.

In the literature, many estimators proposed for linear regression models can be generalized to be applied to PRMs. For example, the estimator depending on the Ridge estimator in linear regression models was proposed by Yang and Chang^29^. In this sense, the biased estimator proposed by Yang and Chang^29^ is adapted from the PRMs by Asar and Genç^15^. In addition, this estimator is applied to Negative Binomial regression models by Huang and Yang^30^. Depending on the PRE, the estimator given by Huang and Yang^30^ in the literature has been as follows:

10 $$\hat{\beta }_{PHY} \left( {k,d} \right) = \left( {X^{\prime}\hat{W}X + I} \right)^{ - 1} \left( {X^{\prime}\hat{W}X + dI} \right)\left( {X^{\prime}\hat{W}X + kI} \right)^{ - 1} X^{\prime}\hat{W}X\hat{\beta }_{MLE} ,\quad k > 0, 0 < d < 1,$$

where *k* and *d* are two biasing parameters. Although the estimator given in (10) is depending on the PRE, it is a general estimator which includes the MLE, PRE, and PLE as special cases, too.

From this point of view, another estimator depending on the Ridge estimator in linear regression models was proposed by Sakallıoğlu and Kaçıranlar^31^, and is defined by Sakallıoğlu and Kaçıranlar^31^ which is defined as:

11 $$\hat{\beta }_{SK} \left( {k,d} \right) = \left( {X^{\prime}X + I} \right)^{ - 1} \left( {X^{\prime}X + \left( {k + d} \right)I} \right)\hat{\beta }_{RE} { ,}\quad k > 0, - \infty < d < \infty ,$$

where *k* and *d* are two biasing parameters and $\hat{\beta }_{RE} = \left( {X^{\prime}X + kI} \right)^{ - 1} X^{\prime}Y$. In this context, we can generalize the (11) estimators suggested for PRMs. Based on the PRE, we can generalize the estimator proposed by Sakallıoğlu and Kaçıranlar^31^ given in (11) as follows:

12 $$\hat{\beta }_{PSK} \left( {k,d} \right) = \left( {X^{\prime}\hat{W}X + I} \right)^{ - 1} \left( {X^{\prime}\hat{W}X + \left( {k + d} \right)I} \right)\hat{\beta }_{PRE} { ,}\quad k > 0, - \infty < d < \infty ,$$

where *k* and *d* are two biasing parameters. In this case, the estimator given in (12) is a general estimator which includes the MLE, PRE and PLE as special cases. Best of our knowledge, no study has been conducted about estimator in (12) for the PRMs.

In PRMs, it is known that the performance of biased estimators proposed as an alternative to MLE is generally affected by the value of the biasing parameter. In general, the methods used for the estimation of biasing parameters have been adapted similarly to those used in linear regression models. On the other hand, the use of estimators with two biasing parameters has become increasingly widespread in recent years. However, one of the most important problems for estimators with two biasing parameters is finding optimal estimates of the biasing parameters is difficult. For this purpose, many iterative techniques have been proposed to estimate these biasing parameters. In these cases, one of the biasing parameters can be estimated depending on the other biasing parameter, or vice versa^15,16,30^. Thus, the idea arises that an unknown functional relationship may exist between these two biasing parameters.

Based on the information mentioned above, our aim in this article is to introduce a new general class of estimators that arises when there is a functional relationship between the biasing parameters. In addition, the proposed general estimator can be defined to specifically include the estimators given by (4), (5), (6), (10) and (12). Thus, this proposed estimator constitutes a general class of estimators like the estimator given in (9). It is a more efficient alternative estimator when compared with the one defined in (9) which can overcome multicollinearity in the PRMs. Another purpose of this article is to compare these two class estimators with a simulation study under some conditions.

The remainder of the article is organized as follows: In "A new general biased estimator", a new biased estimator is defined and some of its properties are given. The superiority of this estimator over the other biased estimators under the matrix mean square error sense are shown in "The superiority of the PRTE in PRMs". In "Determination of function", several rules are proposed to determine the relationship between the biasing parameters. Two separate Monte Carlo simulation studies are executed in "The Monte Carlo simulation studies". In "Numerical example: the aircraft damage data", a real numerical example is provided to evaluate the performances of the proposed biased estimators. Some concluding remarks are given in "Some concluding remarks".

## A new general biased estimator

For PRMs, we can define a new general class of estimators including (4), (5), (6), (10) and (12) estimators based on the PRE estimator as follows:

13 $$\hat{\beta }_{PRTE} = \left( {X^{\prime}\hat{W}X + I} \right)^{ - 1} \left( {X^{\prime}\hat{W}X + g\left( k \right)I} \right)\hat{\beta }_{PRE} , k > 0,$$

where $g\left( k \right)$ is a continuous function of the biasing parameter $k$. When we select $g\left( k \right)$ as a linear function of the biasing parameter *k* such as $g\left( k \right) = ak + b$ where $a,b \in R$, the Poisson Ridge-type estimator (PRTE) is a general estimator which includes the other biased estimators as special cases:

$\hat{\beta }_{PRTE} = \hat{\beta }_{MLE}$ for $g\left( 0 \right) = 1$ where $k = 0$ and $b = 1$.

$\hat{\beta }_{PRTE} = \hat{\beta }_{PRE}$ for $g\left( k \right) = 1$ where $a = 0$ and $b = 1$.

$\hat{\beta }_{PRTE} = \hat{\beta }_{PLE}$ for $g\left( 0 \right) = b$ where $a = 0$ and $b$ corresponds to the biasing parameter *d*.

$\hat{\beta }_{PRTE} = \hat{\beta }_{PHY} \left( {k,d} \right)$ for $g\left( k \right) = b$ where *b* corresponds to the biasing parameter *d*.

$\hat{\beta }_{PRTE} = \hat{\beta }_{PSK} \left( {k,d} \right)$ for $g\left( k \right) = k + b$ where $a = 1$ and *b* corresponds to the biasing parameter *d*.

Note that, the proposed estimator given in (13) is different form the biased estimator given in (9). That is, when we use $\hat{\beta }_{PRE}$ instead of $\hat{\beta }^{*}$ in (9), the resulting estimator $\hat{\beta }_{ILTE(PRE)}$ is given as follows:

14 $$\hat{\beta }_{ILTE(PRE)} = \left( {X^{\prime}\hat{W}X + kI} \right)^{ - 1} \left( {X^{\prime}\hat{W}X + f\left( k \right)I} \right)\hat{\beta }{\kern 1pt}_{PRE} , k > 0,$$

where $f\left( k \right)$ is a continuous function of the biasing parameter $k$. Note that the estimator given in (14) does not exactly correspond to the estimators given by (10) and (12), respectively. To show that the estimators given in (13) and (14) are different estimators, let’s examine the asymptotic scalar mean square error (SMSE) and asymptotic matrix mean square error (MMSE) of these estimators.

In order to obtain the asymptotic SMSE and the asymptotic MMSE of an estimator, we denote $\alpha = Q^{\prime}\beta ,$ $\Lambda { = }diag\left( {\lambda_{1} ,...,\lambda_{p + 1} } \right) = Q^{\prime}\left( {X^{\prime}\hat{W}X} \right)Q,$ where $\lambda_{1} \ge \lambda_{2} \ge \cdots \lambda_{p + 1} > 0$ are the ordered eigenvalues of $X^{\prime}\hat{W}X,  Q$ is the orthogonal matrix whose columns constitute the eigenvectors of $X^{\prime}\hat{W}X$ and the *i*th element of $Q^{\prime}\beta$ is denoted as $\alpha_{j} ,j = 1,2,...,p + 1.$

The asymptotic SMSE and the asymptotic MMSE of an estimator $\hat{\beta } = H\hat{\beta }_{MLE} ,$ where $H$ is an $\left( {p + 1} \right) \times \left( {p + 1} \right)$ matrix, are defined as:

15 $$\begin{aligned} & MSEM\left( {\hat{\beta }} \right) = E\left( {\hat{\beta } - \beta } \right)\left( {\hat{\beta } - \beta } \right)^{\prime } = H\left( {\hat{\beta }_{MLE} - \beta } \right)\left( {\hat{\beta }_{MLE} - \beta } \right)^{\prime } H^{\prime} + \left( {H\beta - \beta } \right)\left( {H\beta - \beta } \right)^{\prime } \\ & SMSE\left( {\hat{\beta }} \right) = E\left( {\hat{\beta } - \beta } \right)^{\prime } \left( {\hat{\beta } - \beta } \right) = \left( {\hat{\beta }_{MLE} - \beta } \right)^{\prime } H^{\prime}H\left( {\hat{\beta }_{MLE} - \beta } \right) + \left( {H\beta - \beta } \right)^{\prime } \left( {H\beta - \beta } \right). \\ \end{aligned}$$

Note that there is a relationship $SMSE\left( {\hat{\beta }} \right) = tr\left( {MMSE\left( {\hat{\beta }} \right)} \right)$ between MMSE and SMSE criteria. Because of the relation of $\alpha = Q^{\prime}\beta$; $\hat{\beta }_{MLE} , \hat{\beta }_{PRE} , \hat{\beta }_{PLE} , \hat{\beta }_{PLTE} , \hat{\beta }_{ILTE}$ and $\hat{\beta }_{PRTE}$ have the same SMSE values as $\hat{\alpha }_{MLE} , \hat{\alpha }_{PRE} , \hat{\alpha }_{PLE} , \hat{\alpha }_{PLTE} , \hat{\alpha }_{ILTE}$ and $\hat{\alpha }_{PRTE}$, respectively.

Using (9), (13) and (14), it is easily computed that

16 $$\begin{aligned} MMSE\left( {\hat{\beta }_{ILTE} } \right) & = Q\left( {\left( {\Lambda + kI} \right)^{ - 1} \left( {\Lambda + f\left( k \right)I} \right)\Lambda^{ - 1} \left( {\Lambda + f\left( k \right)I} \right)\left( {\Lambda + kI} \right)^{ - 1} } \right. \\ & \quad \left. { + \left( {f\left( k \right) - k} \right)^{2} \left( {\Lambda + kI} \right)^{ - 1} \alpha \alpha^{\prime}\left( {\Lambda + kI} \right)^{ - 1} } \right)Q^{\prime} \\ \end{aligned}$$

17 $$\begin{aligned} MMSE\left( {\hat{\beta }_{{ILTE(PRE)}} } \right) & = Q\left( {\left( {\Lambda + kI} \right)^{{ - 1}} \left( {\Lambda + f\left( k \right)I} \right)\left( {\Lambda + kI} \right)^{{ - 1}} \Lambda \left( {\Lambda + kI} \right)^{{ - 1}} \left( {\Lambda + f\left( k \right)I} \right)\left( {\Lambda + kI} \right)^{{ - 1}} } \right. \\ & \quad \left. { + \left( {\Lambda + kI} \right)^{{ - 1}} \left( {f\left( k \right)\Lambda - 2k\Lambda - k^{2} I} \right)\left( {\Lambda + kI} \right)^{{ - 1}} \alpha \alpha ^{\prime } \left( {\Lambda + kI} \right)^{{ - 1}} \left( {f\left( k \right)\Lambda - 2k\Lambda - k^{2} I} \right)\left( {\Lambda + kI} \right)^{{ - 1}} } \right)Q^{\prime } . \\ \end{aligned}$$

18 $$\begin{aligned} MMSE\left( {\hat{\beta }_{PRTE} } \right) & = Q\left( {\left( {\Lambda + I} \right)^{ - 1} \left( {\Lambda + g\left( k \right)I} \right)\left( {\Lambda + kI} \right)^{ - 1} \Lambda \left( {\Lambda + kI} \right)^{ - 1} \left( {\Lambda + g\left( k \right)I} \right)\left( {\Lambda + I} \right)^{ - 1} } \right. \\ & \quad \left. { + \left( {\left( {g\left( k \right) - k - 1} \right)\Lambda - kI} \right)\left( {\Lambda + I} \right)^{ - 1} \left( {\Lambda + kI} \right)^{ - 1} \alpha \alpha^{\prime}\left( {\Lambda + kI} \right)^{ - 1} \left( {\Lambda + I} \right)^{ - 1} \left( {\left( {g\left( k \right) - k - 1} \right)\Lambda - kI} \right)} \right)Q^{\prime}. \\ \end{aligned}$$

Moreover, we can give the SMSE functions of ILTE, ILTE (PRE) and PRTE as follows:

19 $$SMSE\left( {\hat{\beta }_{ILTE} } \right) = \sum\limits_{j = 1}^{p + 1} {\frac{{\left( {\lambda_{j} + f\left( k \right)} \right)^{2} }}{{\lambda_{j} \left( {\lambda_{j} + k} \right)^{2} }}} + \sum\limits_{j = 1}^{p + 1} {\frac{{\left( {f\left( k \right) - k} \right)^{2} \alpha_{j}^{2} }}{{\left( {\lambda_{j} + k} \right)^{2} }}}$$

20 $$SMSE\left( {\hat{\beta }_{ILTE(PRE)} } \right) = \sum\limits_{j = 1}^{p + 1} {\frac{{\left( {\lambda_{j} + f(k)} \right)^{2} \lambda_{j} }}{{\left( {\lambda_{j} + k} \right)^{4} }}} + \sum\limits_{j = 1}^{p + 1} {\frac{{\left( {f(k)\lambda_{j} - 2k\lambda_{j} - k^{2} } \right)^{2} \alpha_{j}^{2} }}{{\left( {\lambda_{j} + k} \right)^{4} }}}$$

21 $$SMSE\left( {\hat{\beta }_{PRTE} } \right) = \sum\limits_{j = 1}^{p + 1} {\frac{{\lambda_{j} \left( {\lambda_{j} + g\left( k \right)} \right)^{2} }}{{\left( {\lambda_{j} + 1} \right)^{2} \left( {\lambda_{j} + k} \right)^{2} }}} + \sum\limits_{j = 1}^{p + 1} {\frac{{\left( {\left( {g\left( k \right) - k - 1} \right)\lambda_{j} - k} \right)^{2} \alpha_{j}^{2} }}{{\left( {\lambda_{j} + 1} \right)^{2} \left( {\lambda_{j} + k} \right)^{2} }}}$$

where the first term is the asymptotic variance and the second term is the squared bias. It should be noted that MMSE and SMSE functions of ILTE (PRE) and PRTE are different. Also, the MMSE and SMSE functions of other existing functions can be obtained according to the appropriate selection of $f\left( k \right)$ and $g\left( k \right)$.

Let $\hat{\beta }_{1}$ and $\hat{\beta }_{2}$ be any two estimators of $\beta$ parameter. Then, $\hat{\beta }_{2}$ is superior to $\hat{\beta }_{1}$ with respect to the MMSE sense if and only if $MMSE\left( {\hat{\beta }_{1} } \right) - MMSE\left( {\hat{\beta }_{2} } \right)$ is a positive definite (pd) matrix. If $MMSE\left( {\hat{\beta }_{1} } \right) - MMSE\left( {\hat{\beta }_{2} } \right)$ is a non-negative definite matrix, then $SMSE\left( {\hat{\beta }_{1} } \right) - SMSE\left( {\hat{\beta }_{2} } \right) \ge 0.$ But, the reverse is not always true^32^.

In order to compare the MMSEs for the above-mentioned biased estimators, we are using the following theorem.

### Theorem 2.1

*Let* $A$ *be a positive definite matrix, namely* $A > 0,$ *and* $c$ *nonzero vector*. *Then,* $A - cc^{\prime}$ *is positive definite matrix iff* $c^{\prime}A^{ - 1} c \le 1$^33^.

## The superiority of the PRTE in PRMs

In this section, we compare the PRTE with the ILTE according to the MMSE criterion. Here, we give a general theorem for comparing estimators with different choices of $g\left( k \right)$ and $f\left( k \right)$ functions. In this way, a general theorem is obtained for comparing the estimators mentioned above in terms of MMSE sense.

The following theorem is given to show the superiority of PRTE over ILTE.

### Theorem 3.1.

*Let be* $k > 0$ *and* $- \lambda_{j} - \frac{{\left( {\lambda_{j} + 1} \right)\left( {\lambda_{j} + f\left( k \right)} \right)}}{{\lambda_{j} }} < g\left( k \right) < - \lambda_{j} + \frac{{\left( {\lambda_{j} + 1} \right)\left( {\lambda_{j} + f\left( k \right)} \right)}}{{\lambda_{j} }}$ *where* *j*=1,2,...,*p*+1. *Then* $MMSE\left( {\hat{\beta }_{ILTE} } \right) - MMSE\left( {\hat{\beta }_{PRTE} } \right) > 0$ *iff*

22 $$\begin{aligned} & bias\left( {\hat{\beta }_{PRTE} } \right)^{\prime } Q\left( {\left( {\Lambda + kI} \right)^{ - 1} \left( {\Lambda + f\left( k \right)I} \right)\Lambda^{ - 1} \left( {\Lambda + kI} \right)^{ - 1} \left( {\Lambda + f\left( k \right)I} \right)} \right. \\ & \quad \left. { - \left( {\Lambda + I} \right)^{ - 1} \left( {\Lambda + g\left( k \right)I} \right)\left( {\Lambda + kI} \right)^{ - 1} \Lambda \left( {\Lambda + kI} \right)^{ - 1} \left( {\Lambda + g\left( k \right)I} \right)\left( {\Lambda + I} \right)^{ - 1} } \right)^{ - 1} Q^{\prime}bias\left( {\hat{\beta }_{PRTE} } \right) < 1 \\ \end{aligned}$$

where $$bias\left( {\hat{\beta }_{PRTE} } \right) = \left( {\left( {g\left( k \right) - k - 1} \right)\Lambda - kI} \right)Q\left( {\Lambda + I} \right)^{ - 1} \left( {\Lambda + kI} \right)^{ - 1} \alpha$$.

### Proof

Using (19) and (21), we obtain

$$\begin{aligned} & MMSE\left( {\hat{\beta }_{ILTE} } \right) - MMSE\left( {\hat{\beta }_{PRTE} } \right) = Q\left( {\left( {\Lambda + kI} \right)^{ - 1} \left( {\Lambda + f\left( k \right)I} \right)\Lambda^{ - 1} \left( {\Lambda + kI} \right)^{ - 1} \left( {\Lambda + f\left( k \right)I} \right)} \right. \\ & \quad \left. { - \left( {\Lambda + I} \right)^{ - 1} \left( {\Lambda + g\left( k \right)I} \right)\left( {\Lambda + kI} \right)^{ - 1} \Lambda \left( {\Lambda + kI} \right)^{ - 1} \left( {\Lambda + g\left( k \right)I} \right)\left( {\Lambda + I} \right)^{ - 1} } \right)^{ - 1} Q^{\prime} - bias\left( {\hat{\beta }_{PRTE} } \right)bias\left( {\hat{\beta }_{PRTE} } \right)^{\prime } \\ & \quad = Q diag\left\{ {\frac{{\left( {\lambda_{j} + f\left( k \right)} \right)^{2} }}{{\left( {\lambda_{j} + k} \right)^{2} \lambda_{j} }} - \frac{{\lambda_{j} \left( {\lambda_{j} + g\left( k \right)} \right)^{2} }}{{\left( {\lambda_{j} + 1} \right)^{2} \left( {\lambda_{j} + k} \right)^{2} }}} \right\}_{j = 1}^{p + 1} Q^{\prime} - bias\left( {\hat{\beta }_{PRTE} } \right)bias\left( {\hat{\beta }_{PRTE} } \right)^{\prime } . \\ \end{aligned}$$

$D = \left( {\Lambda + kI} \right)^{ - 1} \left( {\Lambda + f\left( k \right)I} \right)\Lambda^{ - 1} \left( {\Lambda + kI} \right)^{ - 1} \left( {\Lambda + f\left( k \right)I} \right) - \left( {\Lambda + I} \right)^{ - 1} \left( {\Lambda + g\left( k \right)I} \right)\left( {\Lambda + kI} \right)^{ - 1} \Lambda \left( {\Lambda + kI} \right)^{ - 1} \left( {\Lambda + g\left( k \right)I} \right)\left( {\Lambda + I} \right)^{ - 1}$ is the pd matrix if $\left( {\lambda_{j} + 1} \right)^{2} \left( {\lambda_{j} + f\left( k \right)} \right)^{2} - \lambda_{j}^{2} \left( {\lambda_{j} + g\left( k \right)} \right)^{2} > 0.$ Thus *D* is the pd matrix if $- \lambda_{j} - \frac{{\left( {\lambda_{j} + 1} \right)\left( {\lambda_{j} + f\left( k \right)} \right)}}{{\lambda_{j} }} < g\left( k \right) < - \lambda_{j} + \frac{{\left( {\lambda_{j} + 1} \right)\left( {\lambda_{j} + f\left( k \right)} \right)}}{{\lambda_{j} }}$ and $k > 0$ where *j*=1,2,...,*p*+1. By Theorem 2.1, the proof is completed.

## Determination of $g\left( k \right)$ function

Since the performance of the biased estimators is related to the choice of biasing parameters, it is an important problem to find the optimal biasing parameters for the proposed biased estimators. Different techniques for estimating the biasing parameters in the PRE, PLE, PLTE, PSK and PHY are generalized depending on the similarities between linear regression models and PRMs^5,15–19,23,30,34^. The performance of PRTE depends on the function $g\left( k \right)$, and therefore only on the biasing parameter $k$. It should be noted that we have given the appropriate choice of the $g\left( k \right)$ function in the introduction that different estimators can be obtained. We may give a method to find the optimal $g\left( k \right)$ function that approximately minimizes $SMSE\left( {\hat{\beta }_{PRTE} } \right)$ according to $k$. Our aim here is to determine the *k* and $g\left( k \right)$ functions together, which can make the $SMSE\left( {\hat{\beta }_{PRTE} } \right)$ function approximately minimum. In other words, our goal here is to choose the appropriate *k* and $g\left( k \right)$ functions such that the decrease in the variance term is greater than the increase in squared bias. Note that $SMSE\left( {\hat{\beta }_{PRTE} } \right)$ is a nonlinear function of the biasing parameter $k$. So, writing $h\left( k \right) = SMSE\left( {\hat{\beta }_{PRTE} } \right),$ then we find $h^{\prime}\left( k \right)$ as follows differentiating $h\left( k \right)$ with respect to $k,$

$$h^{\prime}\left( k \right) = \sum\limits_{j = 1}^{p + 1} {\frac{{2\lambda_{j} \left( {\lambda_{j} - g^{\prime}\left( k \right)\lambda_{j} - g^{\prime}\left( k \right)k + g\left( k \right)} \right)\left[ {\alpha_{j}^{2} \left( {\left( {k + 1 - g\left( k \right)} \right)\lambda_{j} + k} \right) - \left( {\lambda_{j} + g\left( k \right)} \right)} \right]}}{{\left( {\lambda_{j} + 1} \right)^{2} \left( {\lambda_{j} + k} \right)^{3} }}}.$$

When $h^{\prime}\left( k \right) = 0$, there are two facts as follows;

### Fact 1

$\lambda_{j} \left( {\lambda_{j} - g^{\prime}\left( k \right)\lambda_{j} - g^{\prime}\left( k \right)k + g\left( k \right)} \right) = 0$ differential equation is found. From the solution of this differential equation, we obtain

23 $$g\left( k \right) = ck + \left( {c - 1} \right)\lambda_{j} ,$$

where $c$ is the constant of integration.

### Fact 2

$\alpha_{j}^{2} \left( {\left( {k + 1 - g\left( k \right)} \right)\lambda_{j} + k} \right) - \left( {\lambda_{j} + g\left( k \right)} \right) = 0$ equation is found. We have

24 $$\begin{array}{*{20}c} {g\left( k \right) = \frac{{\alpha_{j}^{2} \left( {\lambda_{j} + 1} \right)}}{{1 + \lambda_{j} \alpha_{j}^{2} }}k + \frac{{\left( {\alpha_{j}^{2} - 1} \right)}}{{1 + \lambda_{j} \alpha_{j}^{2} }}\lambda_{j} } & {or} & {g\left( k \right) = \frac{{\alpha_{j}^{2} \left( {\lambda_{j} + 1} \right)}}{{1 + \lambda_{j} \alpha_{j}^{2} }}k + \left( {\frac{{\alpha_{j}^{2} \left( {\lambda_{j} + 1} \right)}}{{1 + \lambda_{j} \alpha_{j}^{2} }} - 1} \right)\lambda_{j} } \\ \end{array} .$$

According to the first and the second facts, it is convenient to choose $g\left( k \right)$ as a linear function of the biasing parameter *k*. Note that, $g\left( k \right)$ which is obtained in Fact 2 is a solution of the differential equation which is obtained in Fact 1. According to the results obtained in Fact 1 and Fact 2, we can propose the following generalizations. Firstly, note that the function $g\left( k \right)$ given in (23) and (24) makes the $SMSE\left( {\hat{\alpha }_{PRTE} } \right)$ function approximately minimum for a *j* value. So, $g\left( k \right)$ depends on the eigenvalues of $X^{\prime}WX$, the unknown parameter $\alpha$ and the estimate of the biasing parameter *k*. In other words, many functions can be determined depending on the functional relationship given in (23) and (24). For example, the following functional relationships can be proposed for the determination of function $g\left( k \right)$:

25 $$g_{1} \left( k \right) = c_{1} k + \left( {c_{1} - 1} \right)\lambda_{\min } \,{\text{where}}\,c_{1} \in \left( {0,1} \right),$$

26 $$g_{2} \left( k \right) = \frac{{\alpha_{\min }^{2} \left( {1 + \lambda_{\min } } \right)}}{{1 + \lambda_{\max } \alpha_{\max }^{2} }}k + \left( {\frac{{\alpha_{\min }^{2} \left( {1 + \lambda_{\min } } \right)}}{{1 + \lambda_{\max } \alpha_{\max }^{2} }} - 1} \right)\lambda_{\min } ,$$

27 $$g_{3} \left( k \right) = \frac{{\min \left( {\alpha_{j}^{2} \left( {\lambda_{j} + 1} \right)} \right)}}{{n\max \left( {1 + \lambda_{j} \alpha_{j}^{2} } \right)}}k + \left( {\frac{{\min \left( {\alpha_{j}^{2} \left( {\lambda_{j} + 1} \right)} \right)}}{{n\max \left( {1 + \lambda_{j} \alpha_{j}^{2} } \right)}} - 1} \right)\lambda_{\min } ,$$

where $\alpha_{\min }^{2}$ and $\alpha_{\max }^{2}$ are defined as the minimum and maximum value of $\alpha_{j}^{2} , j = 1,2,...,p + 1,$ respectively. Similarly, $\lambda_{\min }$ and $\lambda_{\max }$ indicate the minimum and maximum value of the eigenvalue of $X^{\prime}\hat{W}X$, respectively.

In this study, we examined only the first degree polynomial functions given in (25) to (27) for $g\left( k \right)$ function. Note that, the function $g\left( k \right)$ can be selected as any continuous function of the biasing parameter *k*. Therefore, the proposed biased estimator depends on a single biasing parameter *k.* In this case, we should use an appropriate estimate of biasing parameter *k*, which must be estimated to control the conditioning of the $X^{\prime}WX$ matrix. Since the proposed estimator depends on a single biasing parameter *k*, the suitable estimates of *k* can be used given in Månsson and Shukur^18^, Kibria et al.^17^, Algamal^25^. In addition to the previously proposed estimators of the biasing parameter, we can also use the following estimators to estimate *k*:

$$\hat{k}_{PRTE} = \frac{{p\left( {\lambda_{\max } - \lambda_{\min } } \right)}}{n},\hat{k}_{PRTE} = \frac{{\max \left( {\lambda_{j} \hat{\alpha }_{j}^{2} } \right)}}{{\sum\nolimits_{j = 1}^{p + 1} {\hat{\alpha }_{j}^{2} } }},\hat{k}_{PRTE} = \left( {\prod\limits_{j = 1}^{p + 1} {\sqrt {\frac{1}{{\hat{\alpha }_{j}^{2} }}} } } \right)^{{\frac{1}{p + 1}}}$$

where $$m_{j} = \sqrt {\frac{{\hat{\sigma }^{2} }}{{\hat{\alpha }_{j}^{2} }}} ,j = 1,2,...,p + 1$$ and $$\hat{\sigma }^{2} = \frac{1}{n - p - 1}\sum\limits_{i = 1}^{n} {\left( {y_{i} - \hat{y}_{i} } \right)^{2} }$$.

## The Monte Carlo simulation studies

In this section, we designed two simulation schemes to compare the performances of different biased estimators in the PRMs. In the first simulation scheme, we discussed the effects of sample size (*n*), the degree of the collinearity $\left( \rho \right)$ and the number of the explanatory variables $\left( p \right)$ on the performance of the PRTE, PRE, PLE, PLTE, PSK, PHY estimators and PRTE, based on suggested best biasing estimates. In the second simulation design, we examined the effect of the biasing parameter on the performances of the PRTE and ILTE for each set of the values $\left( {n,\rho ,p,\sigma^{2} } \right)$. For both simulation designs, we generated the explanatory variables by following Månsson and Shukur^18^, Kibria et al.^17^, Kibria and Lukman^35^ as

28 $$x_{ij} = \left( {1 - \rho^{2} } \right)^{{{1 \mathord{\left/ {\vphantom {1 2}} \right. \kern-0pt} 2}}} w_{ij} + \rho w_{ip+1} , i = 1,2,..,n, j = 1,2,...,p,$$

where $w_{ij}$ are independent standard normal pseudo-random numbers and $\rho$ is specified such that the correlation between any two variables is given by $\rho^{2}$. Four different sets of correlations are investigated corresponding to $\rho = 0.85,0.9,0.99$ and $0.999$. Number of explanatory variables is determined as $p = 2, 4, 8$ and 12. For each set of explanatory variables, the parameter $\beta$ is selected as the normalized eigenvector corresponding to the largest eigenvalue of $X^{\prime}X$ so that $\beta^{\prime}\beta = 1$. We used glm function in the R Stats package^4^. We also set the intercept term equal to 0.

In the simulation and application sections, the proposed best biasing parameter estimators for PRE, PLE, PLTE, PSK, and PHY estimators are used based on the works of Månsson and Shukur^18^, Månsson et al.^19^, Kibria et al.^17^, Asar and Genç^15^, Alanaz and Algamal^34^, Çetinkaya and Kaçıranlar^16^, Qasim et al.^23^, Huang and Yang^30^.

To estimate *k* in PRE, we used the best estimator of *k* as $\hat{k}_{PRE} = \max \left( {\frac{1}{{m_{j} }}} \right)$ where $m_{j} = \sqrt {\frac{{\hat{\sigma }^{2} }}{{\hat{\alpha }_{j}^{2} }}} ,j = 1,2,...,p$ and $\hat{\sigma }^{2} = \frac{1}{n - p - 1}\sum\nolimits_{i = 1}^{n} {\left( {y_{i} - \hat{\mu }_{i} } \right)^{2} }$ which is recommended by Kibria et al.^17^.

According the results given by Qasim et al.^23^, we used the best estimator of *d* in PLE as $\hat{d}_{PLE} = \max \left( {0,\min \left( {\frac{{\hat{\alpha }_{j}^{2} - 1}}{{\max \left( {\frac{1}{{\lambda_{j} }}} \right) + \hat{\alpha }_{\max }^{2} }}} \right)} \right).$

For PLTE, the biasing parameters *k* and *d* are estimated by grouping them in three different ways as follows:

PLTE I: $\hat{k}_{PLTE} = \max \left( {\frac{1}{{m_{j} }}} \right)$ where $m_{j} = \sqrt {\frac{{\hat{\sigma }^{2} }}{{\hat{\alpha }_{j}^{2} }}} ,j = 1,2,...,p$ and $\hat{d}_{PLTE} = \frac{{\sum\nolimits_{j = 1}^{p} {\frac{{1 - \hat{k}_{PLTE} \hat{\alpha }_{j}^{2} }}{{\left( {\lambda_{j} + \hat{k}_{PLTE} } \right)^{2} }}} }}{{\sum\nolimits_{j = 1}^{p} {\frac{{1 + \lambda_{j} \hat{\alpha }_{j}^{2} }}{{\lambda_{j} \left( {\lambda_{j} + \hat{k}_{PLTE} } \right)^{2} }}} }}$.

PLTE II: $\hat{k}_{PLTE} = \frac{{\lambda_{1} - 100 \lambda_{p} }}{99}$ and $\hat{d}_{PLTE} = \frac{{\sum\nolimits_{j = 1}^{p} {\frac{{1 - \hat{k}_{PLTE} \hat{\alpha }_{j}^{2} }}{{\left( {\lambda_{j} + \hat{k}_{PLTE} } \right)^{2} }}} }}{{\sum\nolimits_{j = 1}^{p} {\frac{{1 + \lambda_{j} \hat{\alpha }_{j}^{2} }}{{\lambda_{j} \left( {\lambda_{j} + \hat{k}_{PLTE} } \right)^{2} }}} }}$.

PLTE III: $\hat{d}_{PLTE} = \frac{1}{2}\min \left\{ {\frac{{\lambda_{j} }}{{1 + \lambda_{j} \hat{\alpha }_{j}^{2} }}} \right\}, j = 1,2,...,p$ and $\hat{k}_{PLTE} = \frac{1}{p}\sum\limits_{j = 1}^{p} {\frac{{\lambda_{j} - \hat{d}_{PLTE}^{*} \left( {1 + \lambda_{j} \hat{\alpha }_{j}^{2} } \right)}}{{\lambda_{j} \hat{\alpha }_{j}^{2} }}}$.

Sakallıoğlu and Kaçıranlar^31^ did not provide a specific technique for estimating the biasing parameters *k* and *d* for SK estimator. Therefore, we used the following estimator to estimate the biasing parameters *k* and *d* in PSK:

PSK: $\hat{k}_{PSK} = \max \left( {\frac{1}{{m_{j} }}} \right)$ where $m_{j} = \sqrt {\frac{{\hat{\sigma }^{2} }}{{\hat{\alpha }_{j}^{2} }}} ,j = 1,2,...,p$ and $\hat{d}_{PSK} = \frac{{\sum\nolimits_{j = 1}^{p} {\frac{{\lambda_{j} \left( {\hat{\alpha }_{j}^{2} - 1} \right)}}{{\left( {\lambda_{j} + 1} \right)^{2} \left( {\lambda_{j} + \hat{k}_{PSK} } \right)^{2} }}} }}{{\sum\nolimits_{j = 1}^{p} {\frac{{\lambda_{j} \left( {1 + \lambda_{j} \hat{\alpha }_{j}^{2} } \right)}}{{\left( {\lambda_{j} + 1} \right)^{2} \left( {\lambda_{j} + \hat{k}_{PSK} } \right)^{2} }}} }}$.

Moreover, we used the methods proposed by Huang and Yang^30^ to estimate the parameters of the PHY estimator. Huang and Yang^30^ proposed two methods. We refer to these methods as (K1, D1) and (K2, D2) (see Huang and Yang^30^ for details). We used these methods by adapting them for the PHY estimator in PRMs. As a result, the estimator obtained with (K1, D1) indicates PHY I, and the estimator obtained with (K2, D2) with PHY II.

We used the following $g\left( k \right)$ functions together with the *k* estimator to determine the PRTE:

PRTE I: $$\hat{k}_{{PRTE {\text{I}}}} = \frac{1}{n}\left( {p\lambda_{\max } - \left( {p + 1} \right)\lambda_{\min } } \right)$$ and $$g\left( k \right) = \frac{{\left( {1 + \lambda_{\min } } \right)\alpha_{\min }^{2} }}{{1 + \lambda_{\max } \alpha_{\max }^{2} }}k + \left( {\frac{{\left( {1 + \lambda_{\min } } \right)\alpha_{\min }^{2} }}{{1 + \lambda_{\max } \alpha_{\max }^{2} }} - 1} \right)\lambda_{\min }$$.

PRTE II: $\hat{k}_{{PRTE {\text{II}}}} = \frac{{p\lambda_{\max } \alpha_{{{\text{med}}}}^{2} }}{{n\alpha_{{{\text{mean}}}}^{2} }}$ and $g\left( k \right) = \frac{{\left( {1 + \lambda_{\max } } \right)\alpha_{\min }^{2} }}{{p\left( {1 + \lambda_{\max } \alpha_{\max }^{2} } \right)}}k + \left( {\frac{{\left( {1 + \lambda_{\max } } \right)\alpha_{\min }^{2} }}{{p\left( {1 + \lambda_{\max } \alpha_{\max }^{2} } \right)}} - 1} \right)\lambda_{\min }$.

PRTE III: $\hat{k}_{{PRTE {\text{III}}}} = \frac{p}{n}\left( {\lambda_{\max } - \lambda_{\min } } \right)$ and $g\left( k \right) = \frac{{\min \left( {\left( {1 + \lambda_{j} } \right)\alpha_{j}^{2} } \right)}}{{n\max \left( {1 + \lambda_{j} \alpha_{j}^{2} } \right)}}k + \left( {\frac{{\min \left( {\left( {1 + \lambda_{j} } \right)\alpha_{j}^{2} } \right)}}{{n\max \left( {1 + \lambda_{j} \alpha_{j}^{2} } \right)}} - 1} \right)\lambda_{\min }$.

PRTE IV: $\hat{k}_{{PRTE {\text{IV}}}} = \frac{{p\max \left( {\lambda_{j} \alpha_{j}^{2} } \right)}}{{n\alpha_{{{\text{mean}}}}^{2} }}$ and $g\left( k \right) = \min \left( {\frac{{\left( {1 + \lambda_{j} } \right)\alpha_{j}^{2} }}{{n\left( {1 + \lambda_{j} \alpha_{j}^{2} } \right)}}} \right)k + \left( {\min \left( {\frac{{\left( {1 + \lambda_{j} } \right)\alpha_{j}^{2} }}{{n\left( {1 + \lambda_{j} \alpha_{j}^{2} } \right)}}} \right) - 1} \right)\lambda_{\min }$. where $\alpha_{{{\text{med}}}}^{2}$ and $\alpha_{{\text{mean}}}^{2}$ are defined as the median end mean value of $\alpha_{j}^{2} , j = 1,2,...,p + 1,$ respectively.

The performance of the estimated MSEs (EMSEs) is used as basis for comparing the proposed estimators which are calculated for an estimator $\hat{\beta }$ of $\beta$ as

29 $$EMSE\left( {\hat{\beta }} \right) = \frac{1}{N}\sum\limits_{r = 1}^{N} {\left( {\hat{\beta }_{r} - \beta } \right)^{\prime } \left( {\hat{\beta }_{r} - \beta } \right)} ,$$

where $\left( {\hat{\beta }_{r} - \beta } \right)$ is the difference between the estimated and true parameter vectors at *r*th replication and *N* is the number of replications. For each case of *n*, *p* and $\rho$, the experiment was replicated 2000 times by generating response variables. Our Monte Carlo simulation studies were conducted using the R Programming Language. The results for different *n*, *p* and $\rho$ are given in Tables 1, 2, 3 and 4 for $p = 2, 4, 8$ and 12 respectively.

**Table 1:** The EMSE values of the estimators when $p = 2.$

*p*	*n*	$\rho$	MLE	PRE	PLE	PLTE I	PLTE II	PLTE III	PHY I	PHY II	PSK	PRTE I	PRTE II	PRTE III	PRTE IV
2	50	0.85	3.3505	0.4251	0.6666	1.6658	1.8154	0.8999	1.8294	1.7538	1.4409	0.3999**	0.4042	0.4000***	0.3997*
2	50	0.9	7.1098	0.3893	0.5615	3.3676	3.5148	0.9444	3.5087	3.4565	2.1748	0.3407***	0.3433	0.3389**	0.3358*
2	50	0.99	32.3787	0.4784	0.3886	14.7461	14.8312	0.6181	14.8231	14.8011	3.2623	0.3428***	0.3434	0.3425**	0.3400*
2	50	0.999	396.6289	0.6843	0.3317	189.2896	189.2943	0.4948	189.2885	189.343	3.2163	0.3250***	0.3252	0.3248**	0.3241*
2	100	0.85	4.1795	0.4290	0.6463	2.0166	2.1865	0.9297	2.1837	2.1022	1.6097	0.3884***	0.3906	0.3863**	0.3856*
2	100	0.9	5.5753	0.4177	0.6092	2.654	2.8457	1.0138	2.8297	2.7448	1.8903	0.3681***	0.3696	0.3653**	0.3643*
2	100	0.99	35.2475	0.5059	0.3983	16.5814	16.7079	0.7410	16.684	16.6501	3.3132	0.3415***	0.3418	0.3411**	0.3396*
2	100	0.999	420.3712	0.7112	0.3395	196.4906	196.4976	0.5258	196.4944	196.5028	2.9325	0.3339***	0.3340	0.3338**	0.3336*
2	200	0.85	4.3705	0.4327	0.6949	2.1726	2.3697	0.9879	2.3652	2.2690	1.7706	0.3982***	0.4007	0.3941**	0.3936*
2	200	0.9	5.3801	0.3963	0.6039	2.6009	2.7865	0.9417	2.7773	2.7027	1.9059	0.3485***	0.3507	0.3450**	0.3444*
2	200	0.99	37.3897	0.5087	0.3891	17.348	17.4741	0.6873	17.4506	17.4194	3.1571	0.3380***	0.3382	0.3377**	0.3368*
2	200	0.999	392.6338	0.7030	0.3369	182.3938	182.3917	0.5019	182.3881	182.3411	2.7437	0.3320**	0.3320**	0.3320**	0.3316*
2	500	0.85	4.6132	0.4175	0.6410	2.2544	2.4440	0.9466	2.4340	2.3525	1.7471	0.3704***	0.3719	0.3668**	0.3667*
2	500	0.9	5.5349	0.4101	0.5948	2.6512	2.8319	0.9460	2.8192	2.7452	1.8788	0.3599***	0.3613	0.3575**	0.3572*
2	500	0.99	43.9818	0.5336	0.3970	20.7216	20.8506	0.7392	20.8297	20.7898	3.3364	0.3490***	0.3491	0.3487**	0.3484*
2	500	0.999	506.1048	0.7193	0.3363	235.1907	235.2138	0.5256	235.2081	235.1556	2.7036	0.3319**	0.3319**	0.3319**	0.3318*

**Table 2:** The EMSE values of the estimators when $p = 4.$

*p*	*n*	$\rho$	MLE	PRE	PLE	PLTE I	PLTE II	PLTE III	PHY I	PHY II	PSK	PRTE I	PRTE II	PRTE III	PRTE IV
4	50	0.85	14.8073	0.3343	0.7139	5.2396	8.1800	1.1858	7.8278	5.7486	4.4187	0.2396**	0.2491***	0.2371*	0.2570
4	50	0.9	14.6284	0.3357	0.7303	5.2304	7.0981	1.1121	7.9068	5.6430	4.5980	0.2350**	0.2411***	0.2338*	0.2434
4	50	0.99	121.3738	0.4260	0.2938	41.7830	42.6074	1.2557	64.1290	45.5313	15.7597	0.1943**	0.1934*	0.1943**	0.1974
4	50	0.999	1772.2454	0.7404	0.2133	605.9529	606.2798	0.4316	927.2069	666.0135	17.0897	0.2062***	0.2053*	0.2061**	0.2061**
4	100	0.85	10.5035	0.3967	0.8442	3.8045	5.2600	1.0593	5.7930	4.0477	3.4920	0.2692***	0.2701	0.2663**	0.2652*
4	100	0.9	17.2789	0.3340	0.6533	6.1263	7.9786	1.1913	9.2264	6.5948	5.1263	0.2315**	0.2341***	0.2301*	0.2350
4	100	0.99	170.7186	0.5055	0.2774	59.7278	60.1472	1.0102	90.0369	64.9378	17.5231	0.2072***	0.2066*	0.2071**	0.2086
4	100	0.999	1151.1888	0.6979	0.2089	387.5967	387.3600	0.4226	607.2847	419.3292	16.4946	0.1994***	0.1986*	0.1994***	0.199**
4	200	0.85	9.8088	0.4143	0.8649	3.5960	5.1733	1.0801	5.4090	3.8168	3.2808	0.2922***	0.2923	0.2883**	0.2880*
4	200	0.9	14.3776	0.3539	0.7104	5.0917	6.5090	1.1040	7.7311	5.4392	4.4110	0.2466***	0.2476	0.2447*	0.2450**
4	200	0.99	159.9949	0.5061	0.2825	55.5575	55.9240	1.0048	84.9881	59.8018	16.3681	0.2116*	0.2121**	0.2116*	0.2140***
4	200	0.999	1301.5585	0.7337	0.2068	450.2752	450.0915	0.4477	688.5009	489.0979	16.2530	0.1975**	0.1974*	0.1975**	0.1975**
4	500	0.85	10.1800	0.3855	0.7937	3.6070	4.9723	1.0087	5.5117	3.8471	3.2249	0.2599	0.2594***	0.2571**	0.2565*
4	500	0.9	15.2034	0.3395	0.6786	5.3519	5.8787	1.1395	8.1412	5.7093	4.5755	0.2242	0.224***	0.2228**	0.2218*
4	500	0.99	148.5091	0.4908	0.2732	50.9870	50.9130	0.9365	78.1364	54.9269	15.5351	0.2013**	0.2013**	0.2013**	0.2010*
4	500	0.999	1818.8245	0.7584	0.2015	632.8561	632.6658	0.4061	962.8215	684.2725	15.6989	0.1942*	0.1943***	0.1942*	0.1945***

**Table 3:** The EMSE values of the estimators when $p = 8.$

*p*	*n*	$\rho$	MLE	PRE	PLE	PLTE I	PLTE II	PLTE III	PHY I	PHY II	PSK	PRTE I	PRTE II	PRTE III	PRTE IV
8	50	0.85	16.9251	0.5693	1.7384	4.9866	9.3544	0.9601	9.9705	5.1974	4.9074	0.2783**	0.3383	0.2766*	0.3358***
8	50	0.9	34.2415	0.3185	1.3459	10.2258	14.8173	1.2638	20.3488	10.5736	9.6449	0.1757**	0.2107***	0.1754*	0.2671
8	50	0.99	376.8382	0.3521	0.2981	112.4872	114.5209	2.4520	222.1473	115.3996	67.5860	0.1270***	0.1186*	0.1268**	0.1344
8	50	0.999	3550.7387	0.5607	0.1043	1032.0721	1032.1650	0.9530	2101.0805	1057.0848	111.6942	0.0985	0.0882**	0.0982***	0.0857*
8	100	0.85	28.0655	0.3338	1.3340	8.1175	11.7144	1.0920	16.4358	8.3348	7.8438	0.1865**	0.2236***	0.1857*	0.2530
8	100	0.9	32.3341	0.3049	1.2851	9.6048	13.0163	1.2299	19.2689	9.7889	9.4039	0.1702**	0.2010***	0.1697*	0.2345
8	100	0.99	364.0779	0.3548	0.2737	107.2006	108.1535	2.2750	217.2630	109.0771	64.2123	0.1104**	0.1102*	0.1104**	0.1236***
8	100	0.999	3532.0028	0.6299	0.1251	1049.2453	1049.0598	0.9829	2116.0276	1068.3102	97.2488	0.1080***	0.1066*	0.1080***	0.1079**
8	200	0.85	27.8812	0.3376	1.3170	8.2821	11.5251	1.0887	16.5757	8.4104	7.9684	0.2047**	0.2381***	0.2038*	0.2594
8	200	0.9	38.4506	0.2743	1.0961	11.4140	13.2997	1.2850	23.0604	11.5321	11.0466	0.1537**	0.1698***	0.1533*	0.1851
8	200	0.99	338.5512	0.3522	0.2783	102.2077	102.7632	2.0431	203.3376	103.3862	64.7562	0.1100*	0.1101**	0.1100*	0.1224***
8	200	0.999	3801.1621	0.6525	0.1217	1104.5663	1104.3255	0.7988	2250.1080	1117.1855	90.6886	0.1074**	0.1067*	0.1074**	0.1074**
8	500	0.85	23.9278	0.3604	1.3955	7.2290	8.7036	0.9510	14.4034	7.2898	7.0294	0.1731**	0.1790***	0.1724*	0.1796
8	500	0.9	36.8726	0.2774	1.1082	11.0530	12.5696	1.1896	22.0890	11.1405	10.6167	0.1557**	0.1638***	0.1552*	0.1693
8	500	0.99	333.0063	0.3596	0.2557	97.2928	97.3577	1.9152	197.5752	98.3202	59.9565	0.1048*	0.1053**	0.1048*	0.1091***
8	500	0.999	3371.3805	0.6615	0.1232	993.3309	993.0760	0.8654	2028.3847	1001.5095	86.1849	0.1064**	0.1062*	0.1064**	0.1066***

**Table 4:** The EMSE values of the estimators when $p = 12.$

*p*	*n*	$\rho$	MLE	PRE	PLE	PLTE I	PLTE II	PLTE III	PHY I	PHY II	PSK	PRTE I	PRTE II	PRTE III	PRTE IV
12	50	0.85	53.4955	0.3015	2.0251	14.5174	20.2989	1.0766	32.7296	15.2581	12.7215	0.1246**	0.1642***	0.1245*	0.2562
12	50	0.9	70.2583	0.2442	1.7893	19.5074	24.4279	1.3157	43.2572	20.2771	16.7916	0.1204*	0.1368***	0.1204*	0.2368***
12	50	0.99	548.3644	0.2280	0.4282	152.4782	155.4904	3.3321	336.1475	156.3263	101.6552	0.0906***	0.0699*	0.0905**	0.0998
12	50	0.999	4667.8801	0.4415	0.1033	1265.9671	1266.5324	2.7146	2861.8004	1295.1068	264.6328	0.0879	0.0658**	0.0875***	0.0628*
12	100	0.85	36.9427	0.4167	2.2049	10.1231	15.1137	0.9488	23.0251	10.3937	10.0361	0.1688**	0.2419***	0.1685*	0.2954
12	100	0.9	48.6854	0.2949	1.9479	13.3593	17.9167	1.0852	30.7732	13.6499	13.3409	0.1201**	0.1766***	0.1200*	0.2466
12	100	0.99	420.8219	0.2308	0.4158	113.6048	115.2467	2.9800	261.4296	115.2886	92.9058	0.0815**	0.0765*	0.0815**	0.1047***
12	100	0.999	5694.2354	0.5262	0.0988	1561.364	1561.328	2.0811	3598.6116	1583.9902	249.9250	0.0753	0.0709*	0.0752***	0.0720**
12	200	0.85	35.8940	0.3973	2.1548	9.9192	14.1292	0.9314	22.5629	10.0764	9.8577	0.1831**	0.2617***	0.1826*	0.2999
12	200	0.9	55.6204	0.2545	1.7140	15.4278	18.5915	1.1692	35.2408	15.5948	15.3354	0.1152**	0.1540***	0.1150*	0.2043
12	200	0.99	498.4333	0.2532	0.3347	136.5324	137.0078	2.8547	312.8818	137.7243	113.3388	0.0745**	0.0737*	0.0745**	0.0897***
12	200	0.999	4934.7958	0.5365	0.1022	1376.3954	1376.3458	1.9218	3132.8827	1388.3893	241.3478	0.0723**	0.0711*	0.0723**	0.0740***
12	500	0.85	36.7089	0.3639	2.0657	10.1674	12.1931	0.8892	23.2058	10.2179	10.1927	0.1459**	0.1713***	0.1455*	0.1818
12	500	0.9	60.5669	0.2356	1.5392	16.9069	18.6269	1.1705	38.3551	16.9884	16.6802	0.1214**	0.1491***	0.1212*	0.1782
12	500	0.99	598.8263	0.2989	0.2946	169.3809	169.6310	3.0236	380.3873	170.2031	126.8035	0.0774*	0.0780**	0.0774*	0.0934***
12	500	0.999	4728.9493	0.5541	0.1029	1325.9854	1325.7740	1.9088	2999.9883	1332.0518	227.6858	0.0733**	0.0732*	0.0733**	0.0750

The bold numbers in the tables show the estimators with the smallest EMSE values, and in addition, the signs (*), (**), and (***) represent the first, second, and third smallest EMSE values in each row, respectively. The results from Tables 1, 2, 3 and 4 are listed below:

1. According to the results from Tables 1, 2, 3 and 4, it can be seen that the degree of correlation $\left( \rho \right),$ the number of explanatory variables $\left( p \right)$ and the sample size $\left( n \right)$ have different effects on all estimators in the simulation.
2. It has been observed that the EMSE values of PRTE I, PRTE II, PRTE III and PRTE IV are smaller than the other existing biased estimators. Although our proposed estimators PRTE I, PRTE II, PRTE III, and PRTE IV outperformed other existing estimators in all cases, it is also observed that they outperformed each other in different $n, p$ and $\rho$ values.
3. When the number of variables *p*, and $\rho$ are kept constant, the number of observations in the model did not have a significant effect on the PRTE I, PRTE II, PRTE III, and PRTE IV.
4. Regardless of *n* and *p* values, it is observed that PRTE I, PRTE II, PRTE III, and PRTE IV tended to give low EMSE values at high correlation.
5. When the number of observations $\left( n \right)$ and correlation $\left( \rho \right)$ in the model are kept constant, the EMSE values for PRTE I, PRTE II, PRTE III, and PRTE IV decrease with the increase in the number of explanatory variables $\left( p \right)$.

In summary, all our proposed estimators outperformed the other considered estimators in all scenarios. However, it can be seen that there are cases where our estimators outperform each other due to different* k* and $f\left( k \right)$ function choices in different scenarios. As a result, we can observe that the number of observations has a relatively small effect on EMSE values compared to $\rho$ and *p*. In other words, PRTEs have a robust structure according to the number of observations, therefore it gives very good results in case of high collinearity.

In the second simulation scheme, we examined the effects of the biasing parameter *k* on ILTEs and PRTE performances when the sample size $\left( n \right)$, degree of the collinearity $\left( \rho \right)$, and number of explanatory variables $\left( p \right)$ are constant. The purpose of this simulation is to examine the performances of ILTE and PRTE at various values of the biasing parameter *k* according to the EMSE values given in (29). The biasing parameter *k* was not estimated in the second simulation scheme. Only the EMSE values obtained by increasing the *k* values in the range $\left[ {0,{ 2}} \right]$ by 0.1 were compared. There are many $f\left( k \right)$ and $g\left( k \right)$ functions we considered to evaluate the performances of these estimators. In order to compare the performances of these estimators under some *n, p* and $\rho$ as an example, the ILTEs and PRTE determined by the following $f\left( k \right)$ and $g\left( k \right)$ functions are considered:

- $$\hat{\beta }_{ILTE} = \left( {X^{\prime}\hat{W}X + kI} \right)^{ - 1} \left( {X^{\prime}\hat{W}X + f\left( k \right)I} \right)\hat{\beta }{\kern 1pt}_{MLE}$$ where $$f\left( k \right) = \frac{{\lambda_{\min } \alpha_{\min }^{2} }}{{1 + \lambda_{\max } \alpha_{\max }^{2} }}k + \left( {\frac{{\lambda_{\min } \alpha_{\min }^{2} }}{{1 + \lambda_{\max } \alpha_{\max }^{2} }} - 1} \right)\lambda_{\min }$$
- $$\hat{\beta }_{{ILTE{ (}PRE)}} = \left( {X^{\prime}\hat{W}X + kI} \right)^{ - 1} \left( {X^{\prime}\hat{W}X + f\left( k \right)I} \right)\hat{\beta }_{PRE}$$ where $$f\left( k \right) = \frac{{\alpha_{\min }^{2} }}{{1 + \lambda_{\max } \alpha_{\max }^{2} }}\left( {k + \lambda_{\min } } \right)^{2} - \left( {k + \lambda_{\min } } \right)$$
- $$\hat{\beta }_{PRTE} = \left( {X^{\prime}\hat{W}X + I} \right)^{ - 1} \left( {X^{\prime}\hat{W}X + g\left( k \right)I} \right)\hat{\beta }_{PRE}$$ where $$g\left( k \right) = \frac{{\left( {\lambda_{\min } + 1} \right)\alpha_{\min }^{2} }}{{1 + \lambda_{\max } \alpha_{\max }^{2} }}k + \left( {\frac{{\left( {\lambda_{\min } + 1} \right)\alpha_{\min }^{2} }}{{1 + \lambda_{\max } \alpha_{\max }^{2} }} - 1} \right)\lambda_{\min }$$

Note that, when we use $\hat{\beta }_{PRE}$ instead of $\hat{\beta }^{*}$ in $\hat{\beta }_{ILTE}$, the obtained estimator is shown $\hat{\beta }_{ILTE(PRE)}$. Also, the $f\left( k \right)$ functions used in the $\hat{\beta }_{ILTE}$ and $\hat{\beta }_{ILTE(PRE)}$ were determined in accordance with the rules given by Akay and Ertan^5^. Note that when the method given by Akay and Ertan^5^ is applied to $\hat{\beta }_{ILTE(PRE)}$, $f\left( k \right)$ that minimizes the $SMSE\left( {\hat{\beta }_{ILTE(PRE)} } \right)$ function is a quadratic function.

**Figure 1:**
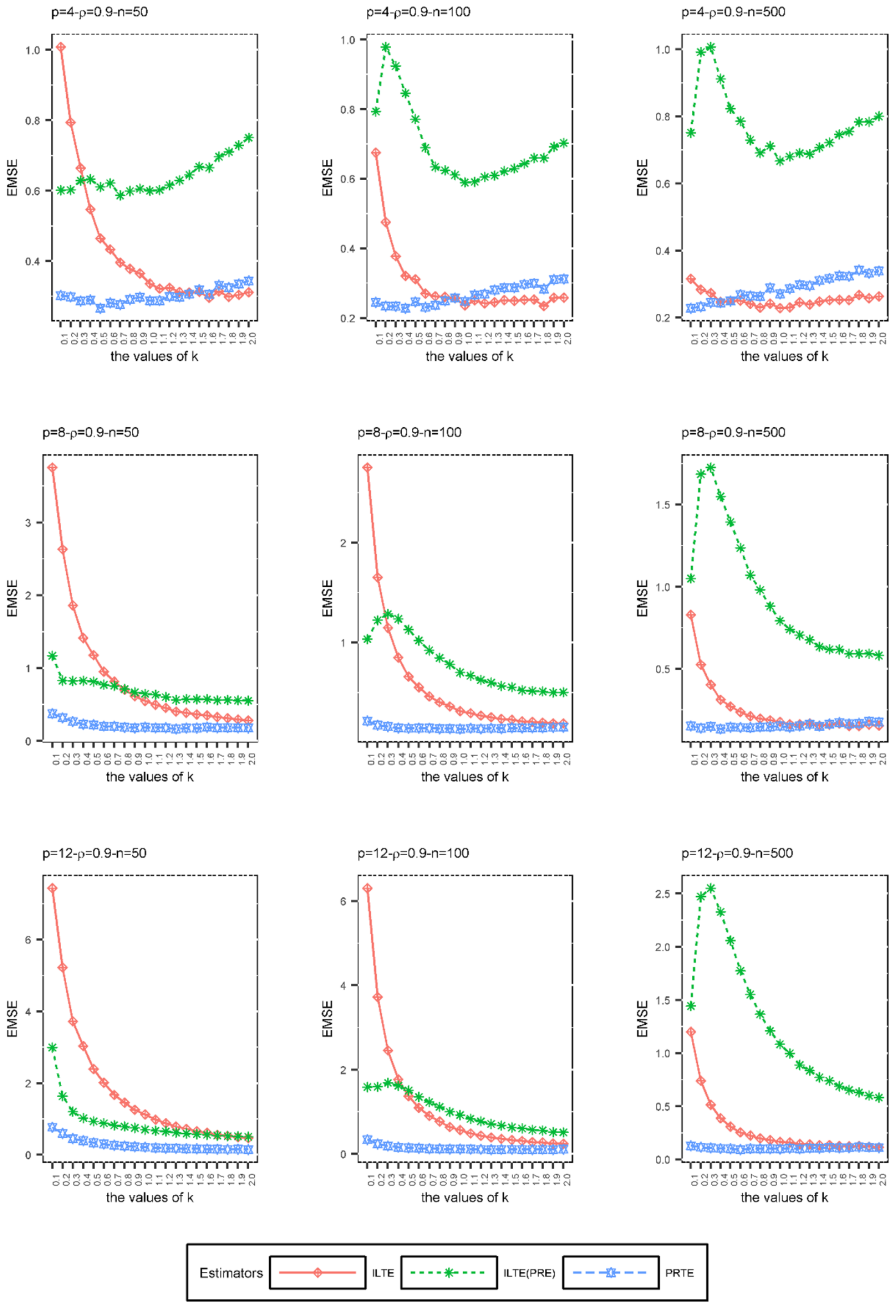
The EMSE values of ILTE, ILTE(PRE), PRTE as a function of *k* values where $\rho = 0.9$.

**Figure 2:**
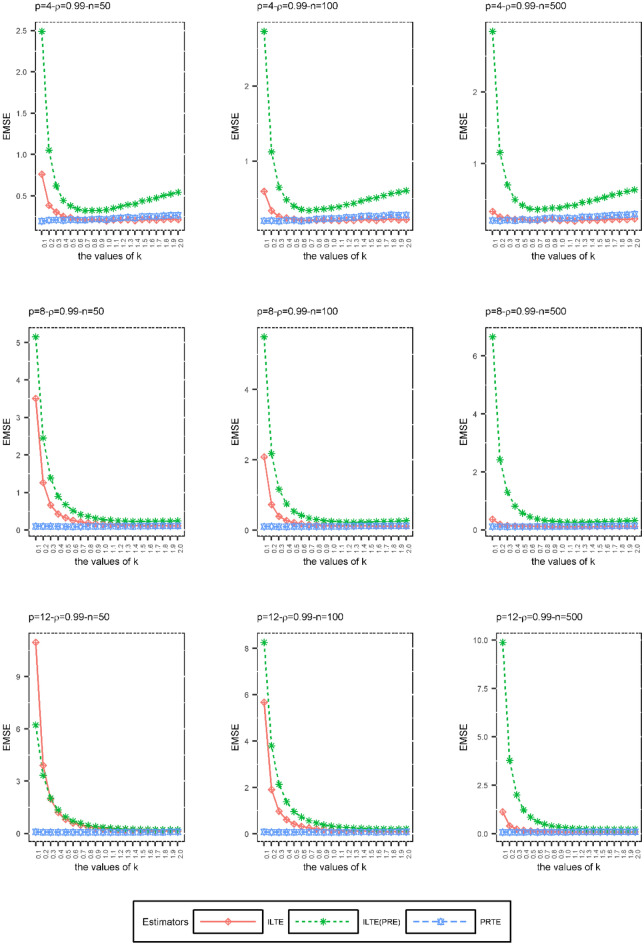
The EMSE values of ILTE, ILTE(PRE), PRTE as a function of* k* values where $\rho = 0.99$.

**Figure 3:**
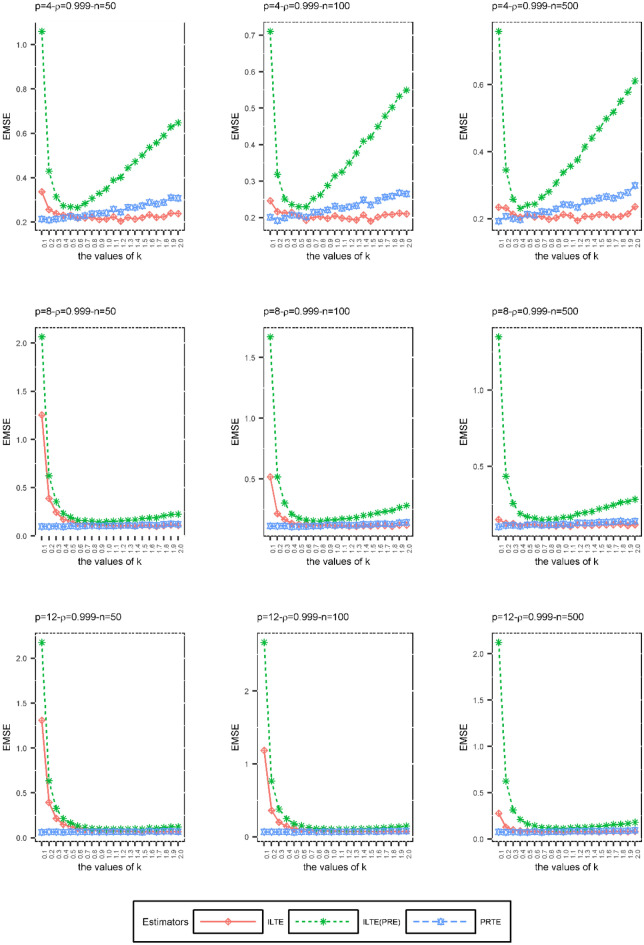
The EMSE values of ILTE, ILTE(PRE), PRTE as a function of* k* values where $\rho = 0.999$.

We considered the cases $\rho = 0.9,0.99, 0.999$, $n = 50,100,500$, and $p = 4,8,12$. Depending on these *n*, $\rho$ and *p* values, the explanatory variables are generated according to (28). The simulation is repeated 2000 times for each *k* value. The results are given graphically in Figs. 1, 2 and 3.

According to Figs. 1, 2 and 3, we can obtain the following results depending on each set of the values $\left( {n,\rho ,p} \right)$;

1. At small values of the biasing parameter *k*, PRTE outperforms other ILTE and ILTE(PRE). Although both the PRTE and ILTE(PRE) include the PRE, the performance of the ILTE(PRE) is quite poor compared to the PRTE at small values of the biasing parameter.
2. When the collinearity between the explanatory variables is relatively low, i.e. $\rho = 0.9$, ILTE(PRE) exhibits quite different behavior from ILTE and PRTE. If the value of correlation of explanatory variables and the number of explanatory variables increases, ILTE, ILTE(PRE) and PRTE show almost the same behavior. However, PRTE exhibits a more consistent behavior at varying values of the biasing parameter* k*.

As a result of the second simulation design, we recommend the PRTE to the researchers. In general, the performance of these estimators depends on $f\left( k \right)$ and $g\left( k \right)$ functions, respectively. In practice, we need to replace these functions with suitable functional relationships that can occur between the biasing parameters.

## Numerical example: the aircraft damage data

In this section, the aircraft damage data is reanalyzed to demonstrate the benefits of PRTE. This data consists of 30 observations with three explanatory variables. The first variable $\left( {x_{1} } \right)$ is a dichotomous variable showing the type of the aircraft. The explanatory variables $\left( {x_{2} } \right)$ and $\left( {x_{3} } \right)$ are bomb load in tons and total months of aircrew experience, respectively. The count variable *y* is the number of locations where damage was inflicted on the aircraft^3^. This dataset is also used by Myers et al.^3^ , Asar and Genç^15^, Amin et al*.*^7^, Lukman et al*.*^36^, and Akay and Ertan^5^.

Asar and Genc^15^, Amin et al.^7^ and Akay and Ertan^5^ considered the following model $\mu = \exp \left( {\beta_{0} + \beta_{1} x_{1} + \beta_{2} x_{2} + \beta_{3} x_{3} } \right)$. Except for the intercept term, the eigenvalues of $X^{\prime}X$ are 208,522.5106, 374.8961 and 4.3333. Thus, the condition number is 48,120.9495, indicating a high multicollinearity problem among the explanatory variables. Firstly, the variables are standardized and then the intercept term is added to the vector of variables. Also, the eigenvalues of the matrix $X^{\prime}WX$ are obtained as $\lambda_{1} = 4{7}{\text{.5850}}$ $\lambda_{2} = {2}{\text{.2844}}$, $\lambda_{3} = {1}{\text{.4097}}$ and $\lambda_{4} = {0}{\text{.3681}}$. The condition number is 129.2719 which is considerably larger than 30, indicating that MLE is still affected due to multicollinearity. The numerical results are given in Tables 5 to compare the PRTEs with other existing estimators.

**Table 5:** The estimated parameter values and the SMSE values of the estimators.

	$\hat{\beta }_{0}$	$\hat{\beta }_{1}$	$\hat{\beta }_{2}$	$\hat{\beta }_{3}$	${\text{var}} \left[ {\hat{\beta }} \right]$	$SMSE\left[ {\hat{\beta }} \right]$
$\hat{\beta }_{MLE}$	0.1262	1.5576	2.6710	− 1.4157	3.8847	
$\hat{\beta }_{PRE}$ $\left( {\hat{k}_{PRE} = 2.7044} \right)$	0.4200	0.7970	1.2620	− 0.5504	0.2329	0.3707
$\hat{\beta }_{PLE}$ $\left( {\hat{d}_{PLE} = 0} \right)$	0.2994	1.1554	1.8704	− 0.8852	0.6714	0.6739
$\hat{\beta }_{{PLTE {\text{I}}}}$ $\left( {\hat{k} = 2.7044, \hat{d} = - 1.2946} \right)$	0.2793	1.1611	1.9365	− 0.9646	1.3472	1.3494
$\hat{\beta }_{{PLTE {\text{II}}}}$ $\left( {\hat{k} = 0.1088, \hat{d} = 0.3268} \right)$	0.2329	1.3552	2.1716	− 1.0640	0.6947	0.6956
$\hat{\beta }_{{PLTE {\text{III}}}}$ $\left( {\hat{k} = 36.3139, \hat{d} = 0.0484} \right)$	0.3820	0.1383	0.2081	− 0.0787	0.0093	0.0556
$\hat{\beta }_{{PHY I}}$ $\left( {\hat{K}_{1} = 0.0637, \hat{D}_{1} = 0.9485} \right)$	0.1507	1.5078	2.5561	− 1.3366	2.8723	2.8723
$\hat{\beta }_{{PHY II}}$ $\left( {\hat{K}_{2} = 0.0506, \hat{D}_{2} = {0}{\text{.2048}}} \right)$	0.2734	1.2170	1.9907	− 0.9645	0.9259	0.9277
$\hat{\beta }_{PSK}$ $\left( {\hat{k}_{SK} = 2.7044, \hat{d}_{SK} = 1.2396} \right)$	0.2117	1.4161	2.3129	− 1.1287	1.1497	1.1502
$$\begin{gathered} \hat{\beta }_{{PRTE {\text{I}}}} \left( {g\left( k \right) = 0.1766 \times 10^{ - 4} k - 0.3681} \right) \hfill \\ \hat{k}_{{PRTE {\text{I}}}} = 6.2833 \hfill \\ \end{gathered}$$	0.5387	0.3238	0.4858	− 0.1687	0.0305	0.0513
$$\begin{gathered} \hat{\beta }_{{PRTE{ {\rm I}{\rm I}}}} \left( {g\left( k \right) = 0.1568 \times 10^{ - 3} k - 0.3680} \right) \hfill \\ \hat{k}_{{PRTE{ {\rm I}{\rm I}}}} = 1.8500 \hfill \\ \end{gathered}$$	0.4952	0.5991	0.9146	− 0.3420	0.0887	0.1011
$$\begin{gathered} \hat{\beta }_{{PRTE{ {\rm I}{\rm I}{\rm I}}}} \left( {g\left( k \right) = 0.1003 \times 10^{ - 4} k - 0.3681} \right) \hfill \\ \hat{k}_{{PRTE{ {\rm I}{\rm I}{\rm I}}}} = 6.2956 \hfill \\ \end{gathered}$$	0.5387	0.3234	0.4852	− 0.1684	0.0305	0.0512
$$\begin{gathered} \hat{\beta }_{{PRTE {\text{IV}}}} \left( {g\left( k \right) = 0.2769 \times 10^{ - 3} k - 0.3680} \right) \hfill \\ \hat{k}_{{PRTE {\text{IV}}}} = 1.2282 \hfill \\ \end{gathered}$$	0.4695	0.6907	1.0597	− 0.4083	0.1198	0.1301

In addition, the bootstrap sampling method is used to calculate the SMSE values of the given biased estimators. For this reason, 10,000 bootstrap samples have been created. For each of these samples, the parameter estimates of the given biased estimators are calculated. The mean of the MLE estimates is considered the real parameters. Then the calculated SMSE values are given in Table 5. From Table 5, it can be seen that the estimator with the best SMSE value is PRTE I and PRTE III.

**Figure 4:**
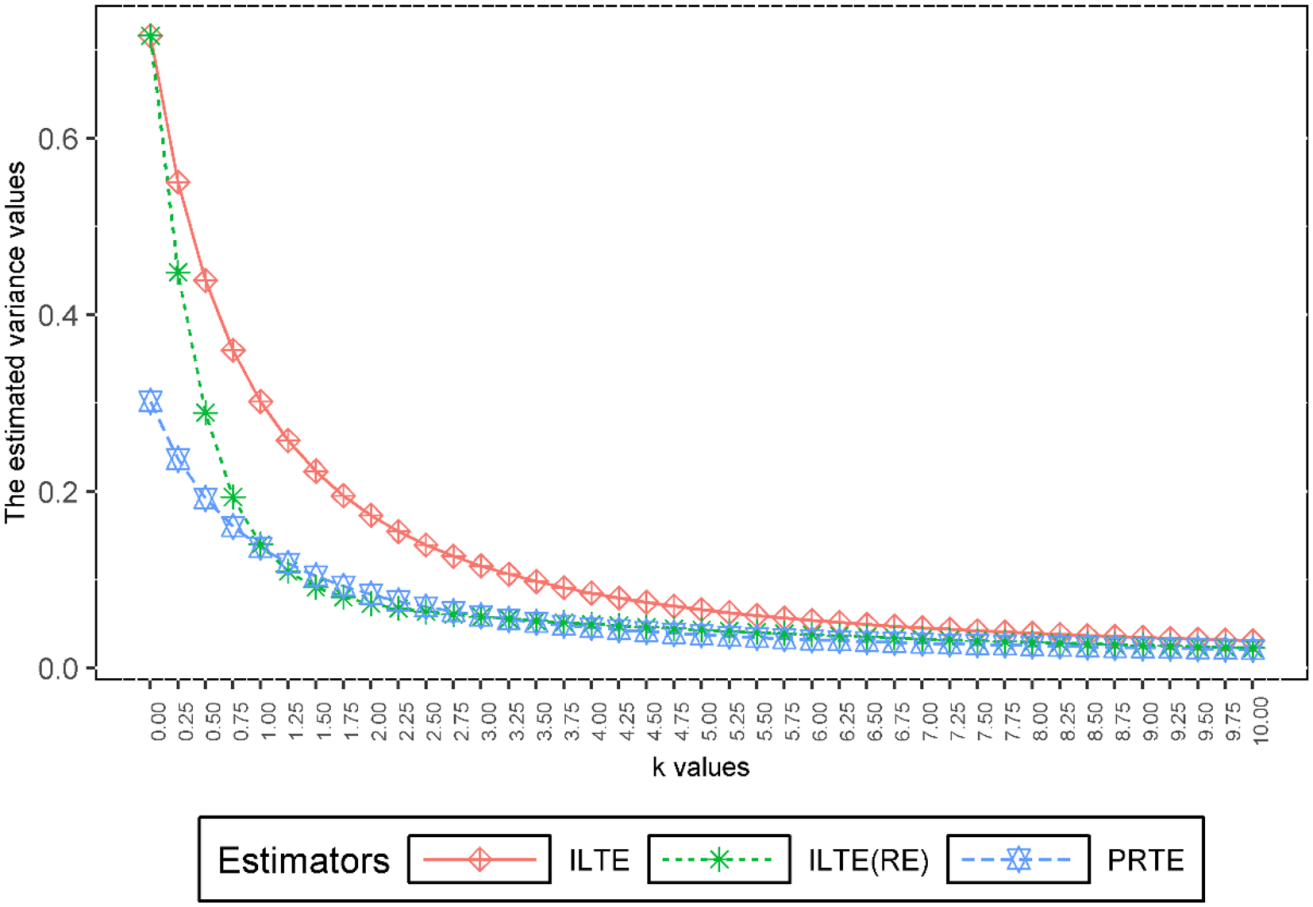
The estimated variance values of ILTE, ILTE(PRE) and PRTE as a function of *k*.

**Figure 5:**
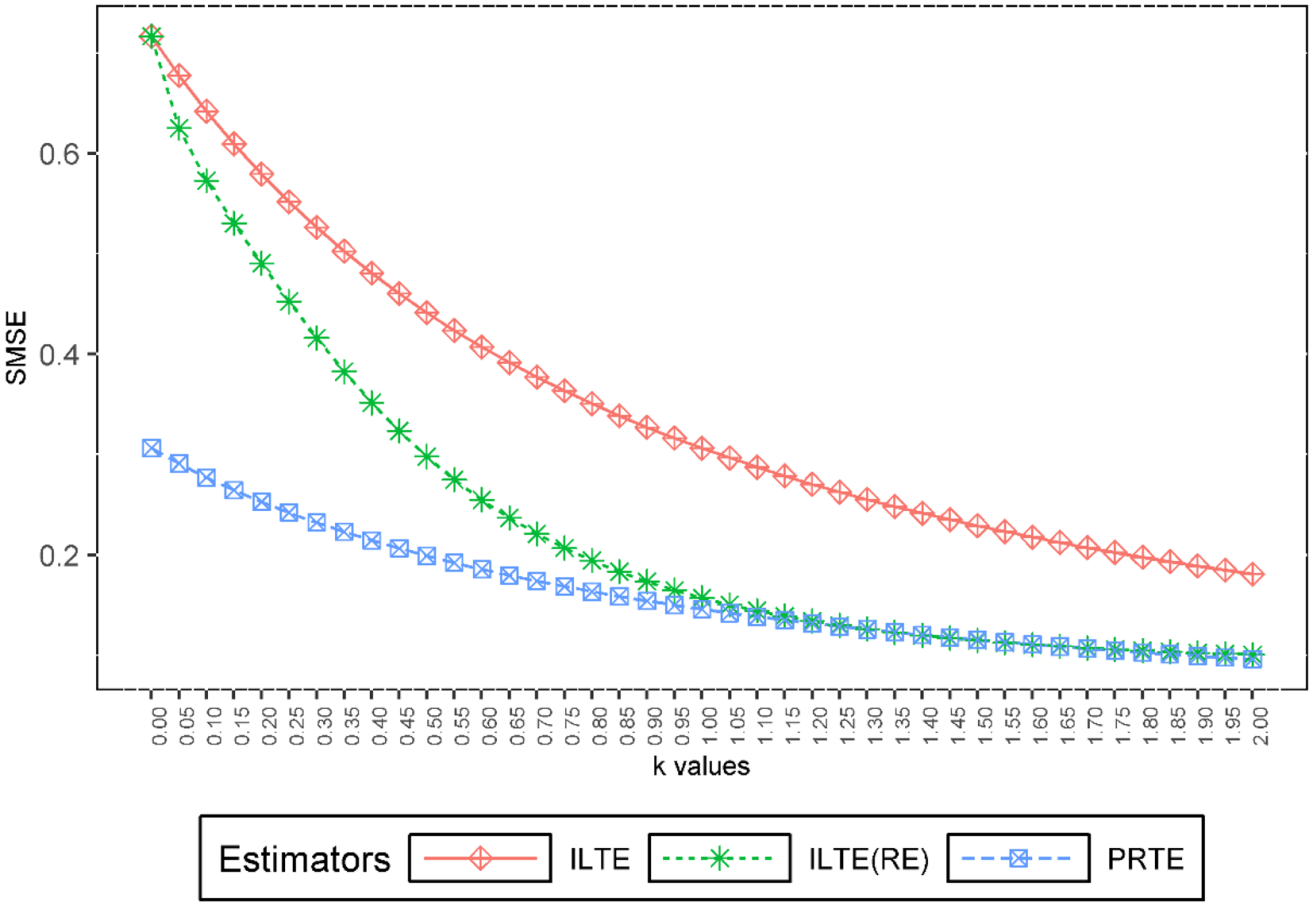
The SMSE values of ILTE, ILTE(PRE) and PRTE as a function of *k*.

Now, we want to examine the performances of ILTE, ILTE(PRE), and PRTE, which were examined in the previous section. Figure 4 graphically shows the estimated variance values of these estimators based on the value of the biasing parameter *k*. Also, Fig. 5 shows the SMSE performance of $\hat{\beta }_{ILTE}$, $\hat{\beta }_{ILTE(PRE)}$ and $\hat{\beta }_{PRTE}$ estimators according to the biasing parameter *k*.

Figures 4 and 5 indicating that the proposed PRTE is a strong alternative to other estimators at small values of the biasing parameter *k*. This result is also compatible with the second simulation results given in the previous section.

To compare the estimators under the MMSE sense, the parameter estimation obtained with the bootstrap sampling method is used in place of the unknown parameter $\alpha$. R Programming is used with tolerance $10^{ - 12}$ to show the MMSE differences as a positive definite (pd) matrix. That is, if any of the eigenvalues is less than or equal to tolerance, then the matrix is not pd. Otherwise, the considered matrix is pd.

Finally, our aim in this part is to compare the estimators obtained from the choice of various $f\left( k \right)$ and $g\left( k \right)$ functions as a result of the theorem given in  “The superiority of the PRTE in PRMs”. To illustrate Theorem 3.1, the function $f\left( k \right)$ and $g\left( k \right)$ are taken as $f\left( k \right) = 0.05k + 0.05$ and $g\left( k \right) = 0.5k - 0.05$, respectively. In this case, ${\text{cov}} \left( {\hat{\beta }_{ILTE} } \right) - {\text{cov}} \left( {\hat{\beta }_{PRTE} } \right)$ is pd matrix for for $0 < k \le 2.0057$. Also, *k* values which provide (22) criterion are $0 < k < 2.0054$. Consequently, $MMSE\left( {\hat{\beta }_{ILTE} } \right) - MMSE\left( {\hat{\beta }_{PRTE} } \right)$ is the pd matrix where $0 < k < 2.0054$.

## Some concluding remarks

In this article, we defined a new general class of estimator named the PRTE as an alternative to MLE and the other existing biased estimators in the presence of multicollinearity for the PRMs. The PRTE is a general estimator which includes other biased estimators, such as the PRE, PLE, PHY and PSK estimators as special cases. In this study, we propose several rules for the determination of function $g\left( k \right)$. By using Monte Carlo simulations, the performance of the proposed PRTE with the existing estimators is evaluated in the smaller EMSE sense. The results show that the proposed PRTE outperforms the existing estimators in case of high multicollinearity. In addition, the comparison of ILTEs and PRTE is given with a general simulation study. In this simulation study, these two general estimators are compared according to the values of the biasing parameter *k*. It is observed that the PRTE is superior at small values of the biasing parameter *k*. Although the PRTE and ILTE(PRE) are both depending on the PRE, the main advantage of PRTE over ILTE(PRE) is that it can minimize the SMSE function with the help of a liner function of the biasing parameter *k*. Also, the estimators are applied to real dataset and it is observed that the results are consistent with simulation study. Depending on the experimental conditions examined, the proposed biased estimator outperforms the other existing biased estimators. Therefore, based on the results of the simulations and example, the PRTEs are recommended to the practitioners when there is multicollinearity problem in the PRMs.

## Data Availability

All data generated or analyzed during this study are used by the given reference in this article. The data analyzed/ generated are available upon the reasonable request by the E. E.
